# cGAS-STING pathway regulated by spatiotemporal heterogeneity of tumor microenvironment and precision therapy strategies in lung cancer

**DOI:** 10.1186/s13046-026-03738-y

**Published:** 2026-05-16

**Authors:** Jing-Ying Ye, Ying Pan, Miao Li, Fang-Qing Shen, Fu-Lu Ren, Hao-Yu Wang, Ming Li, Yun Wang

**Affiliations:** 1 Department of Clinical Pharmacology, School of Pharmacy, China Medical University, No.77 Puhe Road, Shenyang North New Area, Shenyang, Liaoning 110122 China; 2 Department of Laboratory Medicine, The First Hospital of China Medical University, Shenyang, Liaoning China; 3 Department of Urology, Shengjing Hospital of China Medical University, No. 36, Sanhao Street, Heping District, Shenyang, 110004 Liaoning China

**Keywords:** cGAS-STING, Tumor Microenvironment, Spatiotemporal Heterogeneity, Artificial Intelligence, Lung Cancer

## Abstract

The cGAS-STING pathway is a central regulator of innate immunity and exhibits a complex dual function in lung cancer: it can activate anti-tumor immune responses but also promote immune escape and metastasis. This “double-edged sword” effect is highly dependent on the spatiotemporal heterogeneity of the tumor microenvironment (TME), referring to dynamic changes in immune and metabolic features across different regions and disease stages. To optimize existing therapies, overcome resistance, and develop novel treatments to improve patient outcomes, it is crucial to deeply understand the interaction between the TME and the cGAS-STING pathway. This review systematically discusses the pro-tumor and anti-tumor mechanisms of the cGAS-STING pathway in lung cancer; the modulation of cGAS-STING pathway activity by TME spatiotemporal heterogeneity, via the dynamic interplay of distinct cell types and physicochemical gradients; and cGAS-STING-driven precision immunotherapy and artificial intelligence (AI) optimization. Integrating TME spatiotemporal heterogeneity, immunometabolism crosstalk, with cGAS-STING pathway function may provide new insights for improving therapeutic strategies, reversing resistance, and enhancing survival outcomes for lung cancer patients.

**Graphical Abstract:**

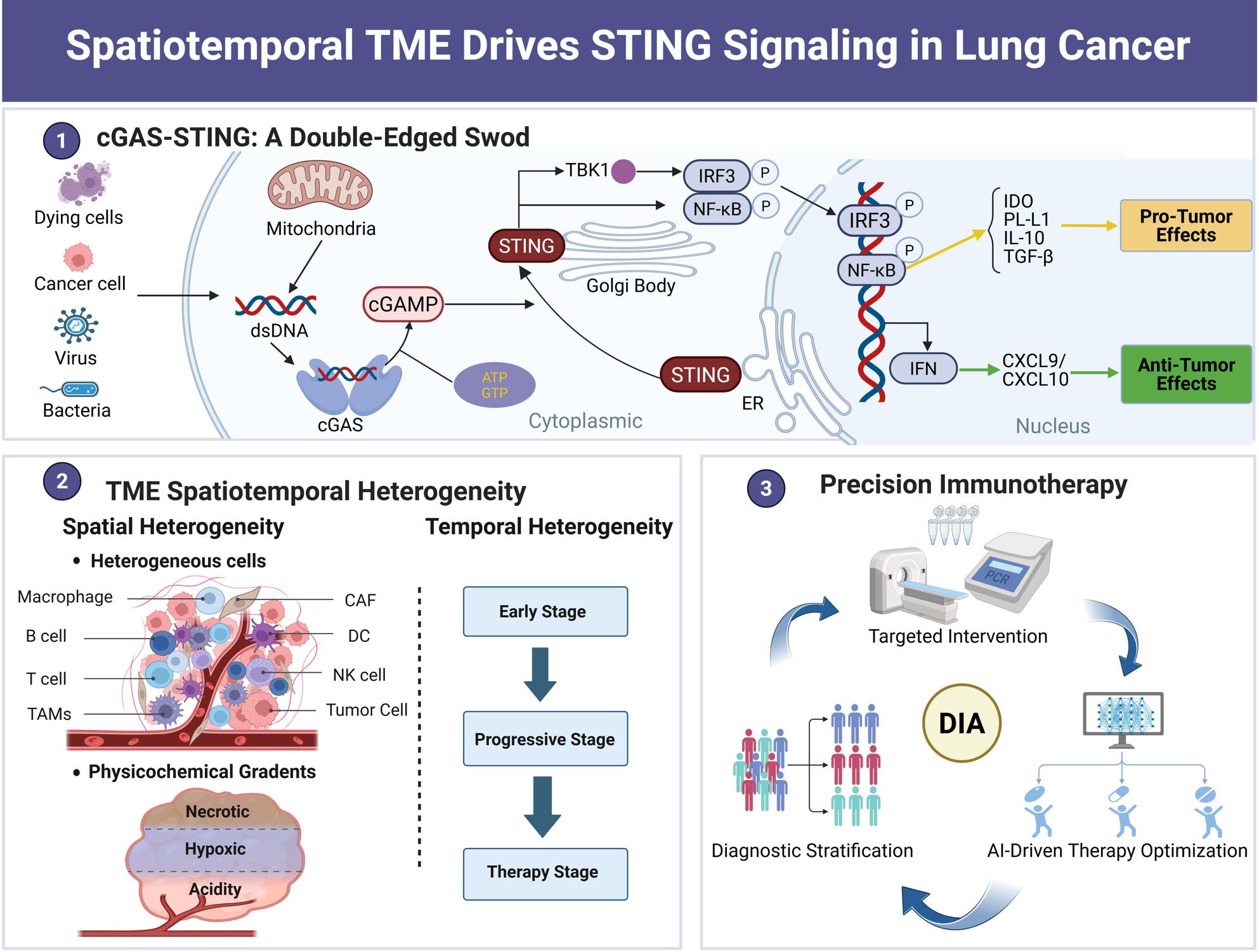

## Background

Globally, the leading cause of cancer-related mortality is attributed to lung cancer [1]. Despite advances in diagnosis and treatment, according to GLOBOCAN data, the global burden of lung cancer in 2022 comprised an estimated 2.5 million incident cases, corresponding to 1.8 million deaths; within this total, China accounted for approximately 1.06 million new cases and 730,000 deaths [2]. The advent of immunotherapy, particularly immune checkpoint inhibitors (ICIs), has marked a paradigm shift in the management of advanced lung cancer, leading to substantially enhanced patient survival [3]. However, challenges remain, including primary and acquired resistance, with approximately 80% of patients not responding initially to ICIs. Tumor heterogeneity, immunosuppression, and therapy resistance are core factors limiting efficacy.

The cGAS-STING pathway constitutes a central axis of innate immune hub that activates downstream type I interferons (IFN-I) and inflammatory cytokine responses by sensing cytosolic DNA, playing an important role in anti-tumor immunity [4]. This pathway mediates immune surveillance through canonical (cGAMP-STING-TBK1-IRF3/NF-κB) and non-canonical (e.g., RNA activation, mtDNA leakage) mechanisms [5]. However, it has a dual role in lung cancer: appropriate activation can enhance anti-tumor immunity, whereas persistent or aberrant activation may lead to immunosuppression and tumor progression [6]. The high heterogeneity of lung cancer results in distinct functional contributions and clinical implications of the cGAS-STING pathway across different subtypes. This discrepancy is particularly evident between non-small cell lung (NSCLC) cancer and small cell lung cancer (SCLC), which exhibit markedly different biological behaviors and treatment strategies [7, 8].

The high heterogeneity in lung cancer is associated with variations in the tumor microenvironment (TME). The TME is a dynamic network composed of diverse cell types, non-cellular components, and soluble factors that influences disease trajectory and treatment outcomes [9]. The spatiotemporal dynamics of the pathway critically dictate its ultimate biological outcome. Based on immune infiltration, tumors can be classified into “cold,” “hot,” and immune-excluded phenotypes, the cGAS-STING pathway serves as a key driver of the immunologically cold-to-hot transition in tumors [10]. Currently, the regulatory mechanisms of TME spatiotemporal heterogeneity on the cGAS-STING pathway are not fully explored. Therefore, deciphering how the dynamic properties of the TME precisely regulate this pathway is essential for developing novel therapeutic strategies.

## The “double-edged sword” of the cGAS-STING signaling pathway in lung cancer

### Overview of the cGAS-STING signaling pathway

**Fig. 1:**
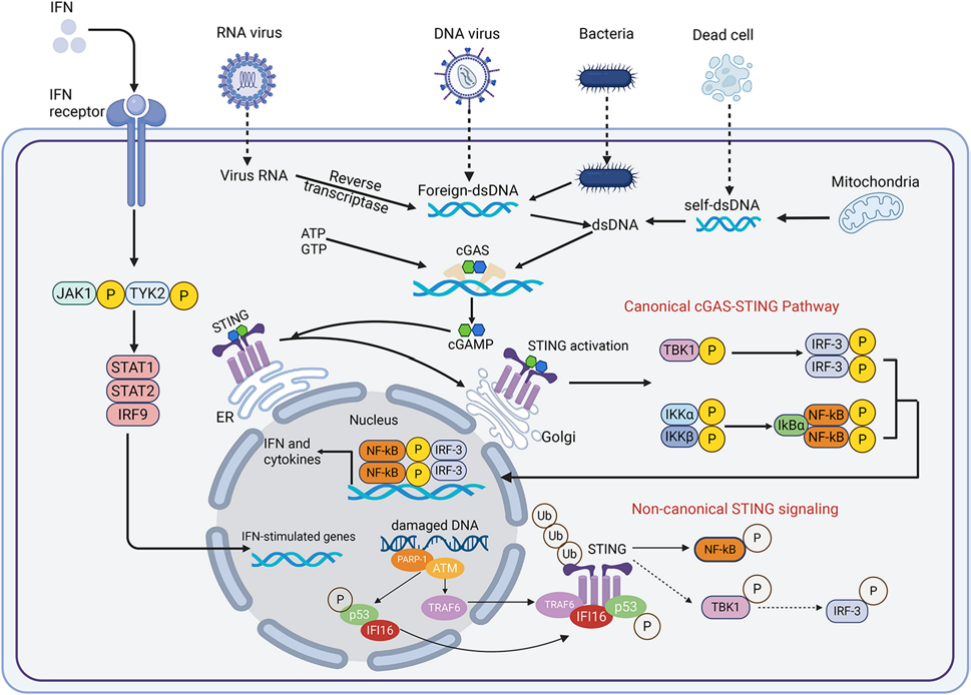
The dual role of the cGAS–STING pathway in lung cancer. This diagram depicts the canonical cGAS–STING signaling pathway and its context-dependent outcomes. Cytosolic DNA activates cGAS to generate the second messenger cGAMP, which stimulates STING–TBK1 signaling, leading to IRF3/NF-κB activation and subsequent production of type I interferons and inflammatory cytokines. Balanced activation promotes antitumor immunity, whereas chronic or dysregulated signaling can drive immunosuppression and tumor progression, illustrating the pathway’s dual function in lung cancer

The cGAS-STING signaling pathway is a crucial innate immune pathway for the body to resist pathogen infection and maintain cellular homeostasis. It functions as an intracellular DNA sensor [11]. Upon recognition of pathogenic DNA or aberrant self-DNA [12], cGAS synthesizes the second messenger cGAMP, which activates STING and recruits TBK1. This subsequently leads to the phosphorylation of IRF3 and NF-κB, inducing the expression of IFN-I and inflammatory factors [13]. In lung cancer, genomic instability provides a persistent source of double-stranded DNA (dsDNA) ligands, yet the effects of pathway activation exhibit significant duality [14] (Fig. 1).

### Anti-tumor effects

The anti-tumor role of the cGAS-STING pathway is primarily achieved through its potent immunostimulatory capacity. The production of IFN-I is the central link through which the cGAS-STING pathway exerts its anti-tumor effects [15]. IFN-I can bind to the interferon-α/β receptor (IFNAR) on the surface of various immune cells within the tumor microenvironment (TME), initiating a cascade of downstream signaling [16]. IFN-I enhances antigen presentation [17], activates killer cells, and promotes T-cell responses. Furthermore, cGAS-STING pathway activation stimulates robust chemokine secretion, facilitating immune cell recruitment and tumor infiltration. This process transforms immunologically “cold” tumors into “hot” ones—a key mechanism for remodeling the tumor immune microenvironment [18]. Beyond indirectly killing tumor cells via the immune system, excessive or intense activation of the cGAS-STING pathway can, under certain circumstances, directly lead to tumor cell death. Studies have shown that in specific types of cancer cells, strong STING activation can induce a form of cell death dependent on the autophagy mechanism [19]. Additionally, some secreted molecules produced upon pathway activation possess intrinsic direct cytotoxicity and can induce tumor cell apoptosis [20]. The cGAS-STING pathway is also recognized to induce a senescent state—a stable cell cycle arrest—in tumor cells, thereby limiting tumor expansion.

### Pro-tumor effects

Research indicates that in various cancers, tumors can exploit the persistent activation of the cGAS-STING pathway and reprogram its downstream signaling to drive phenotypes conducive to tumor growth, metastasis, and drug resistance. Low-level, sustained chronic activation often leads to immune tolerance and immunosuppression [21]. When the cGAS-STING pathway is in a state of prolonged, chronic activation, chronic exposure to type I interferon signaling can induce T-cell exhaustion, characterized by the sustained high expression of inhibitory receptors (e.g., PD-1) on T cells and loss of effector function. Chronic STING activation can upregulate the expression of key immunosuppressive molecules, such as indoleamine 2,3-dioxygenase (IDO). Evidence indicates that chronic activation of the STING pathway can recruit regulatory T cells (Tregs), myeloid-derived suppressor cells (MDSCs), and regulatory B cells (Bregs) [22]. These cells, through the secretion of inhibitory cytokines or cell contact-dependent mechanisms, collectively construct a potent immunosuppressive network, allowing tumors to evade immune surveillance [23]. Apart from indirectly promoting tumor progression by remodeling the TME, aberrant activation of the cGAS-STING pathway can also exert direct effects. Chronic activation of STING tends to favor the activation of the non-canonical NF-κB signaling pathway [24]. Activation of the non-canonical NF-κB pathway is closely associated with cell proliferation, anti-apoptosis, and inflammatory responses, directly promoting tumor cell survival and growth. Tumor cells with high metastatic potential can transfer cGAMP to surrounding stromal cells through various means, activating the STING pathway in these cells and causing them to produce inflammatory factors. These factors, in turn, act back on the tumor cells, activating STAT1 and NF-κB signaling, which greatly enhances the tumor cells’ survival, proliferation, and chemotherapy resistance [25]. Studies suggest that nuclear cGAS may compete with key DNA repair proteins, such as PARP1, for binding to DNA damage sites, thereby inhibiting DNA repair and exacerbating genomic instability. This may drive tumor cells to accumulate more mutations that promote malignant progression, creating a vicious cycle. Furthermore, in some cancers induced by viruses (e.g., HPV, human papillomavirus), the viruses have evolved strategies to suppress host innate immunity for their own survival and replication [26]. Studies have found that in NSCLC, the cGAS-STING pathway is functionally intact but exhibits significant individual variability in expression and activity, critically influencing disease course and treatment response [27]. In contrast, STING expression in SCLC is frequently downregulated or absent, severely compromising IFN-I production and adaptive immunity [28]. 

This histological divergence carries notable clinical implications: the therapeutic potential of STING agonists may be inherently limited in SCLC due to the absence of a functional target, whereas NSCLC patients with intact pathway activity may represent a population more likely to benefit. Conversely, in NSCLC subsets characterized by chronic, low-level STING activation, a state that may contribute to immunosuppression, alternative strategies, such as combination with immune checkpoint inhibitors or intermittent dosing schedules, may be required to mitigate the risk of pro-tumorigenic outcomes [29]. Collectively, these observations suggest that future clinical development of STING targeted therapies should incorporate histology-based stratification, with distinct strategies tailored for NSCLC and SCLC rather than a uniform approach.

## TME spatiotemporal heterogeneity in cGAS-STING signaling

The cGAS-STING pathway is a sharp “double-edged sword”. Whether its ultimate effect is pro-tumorigenic or anti-tumorigenic largely depends on the context of its activation, namely, the overall state of the TME. Intra-tumor heterogeneity (ITH) in human cancers is critical for tumor progression, therapy response, and resistance [30]. Tumor cells, immune cells, stromal cells, vascular networks, the extracellular matrix, and a plethora of signaling molecules and metabolites together form the complex ecosystem of the TME. This ecosystem is not homogeneous but is replete with spatial and temporal heterogeneity [31]. These two forms of heterogeneity fundamentally determine the specific function of the cGAS-STING pathway at a given location and timey [32]. This review will systematically dissect how the spatiotemporal heterogeneity of the TME precisely shapes the functionality of the cGAS-STING pathway by regulating factors such as cell types, signal transduction, and metabolic states.

### Spatial heterogeneity

Tumor tissue is not a uniform mass of cells, but a complex structure composed of distinct functional regions [33]. From the hypoxic, necrotic tumor core to the vascular-rich, immune-cell-active invasive margin, and to distant metastatic sites, significant differences exist in cellular composition, oxygen and nutrient supply, pH, and more. This spatial heterogeneity provides the conditions for the differential activation and functional output of the cGAS-STING pathway [34].

#### Spatial distribution of cell types

The cGAS-STING pathway is expressed across nearly all cell types within the TME, yet the biological outcome of its activation is highly context-dependent and varies dramatically between the tumor cell-intrinsic compartment and the immune/stromal compartment [35]. To dissect this functional dichotomy, we separately discuss the roles of STING signaling in tumor cells versus its effects in immune and stromal cells.

##### Tumor cell-intrinsic compartment

In tumor cells, the consequence of STING activation is inherently dualistic. On one hand, endogenous STING signaling in genomically unstable tumor cells can serve as an immune surveillance mechanism. Upon sensing cytosolic dsDNA derived from micronuclei or DNA damage, tumor cells produce type I interferons (IFN-I) and chemokines, which recruit and activate dendritic cells (DCs) and CD8⁺ T cells, effectively acting as a “self-destruct” signal that facilitates anti-tumor immunity [36]. On the other hand, tumor cells can exploit persistent or low-intensity STING activation to drive pro-tumorigenic phenotypes. Chronic STING signaling tends to favor the non-canonical NF-κB pathway over the IRF3 axis, leading to the secretion of pro-inflammatory and pro-survival factors that enhance tumor cell proliferation, resistance to apoptosis, and metastatic capacity [24, 37]. Additionally, tumor-derived cGAMP can be transferred to neighboring immune cells via exosomes or gap junctions, indirectly shaping the TME in a paracrine manner. Thus, within the tumor cell compartment, STING signaling represents a “double-edged sword” whose net effect depends on activation intensity, duration, and the cellular context [38].

##### Immune and stromal cell compartment

In contrast to tumor cells, STING activation in immune and stromal cells predominantly influences TME through modulating immune cell function, inflammatory tone, and structural remodeling [39, 40]. 

Dendritic Cells (DCs) are central to initiating anti-tumor immunity [41]. Upon sensing DNA from dead tumor cells via cGAS, DCs are potently activated and produce abundant IFN-I. This IFN-I not only enhances DC maturation and antigen-presenting capacity but also enables them to efficiently cross-present captured tumor-associated antigens (TAAs) to naïve CD8⁺T cells, thereby priming a population of cytotoxic T lymphocytes (CTLs) that specifically target tumor cells [42]. This positions DCs as key mediators of STING-driven immune activation.

Tumor-Associated Macrophages (TAMs) exhibit a context-dependent response. In classically activated M1-type macrophages or newly differentiated antigen-presenting cells, STING activation can induce the production of pro-inflammatory cytokines, thereby promoting anti-tumor immunity [43]. Conversely, in tumor-associated macrophages or alternatively activated M2-type macrophages, STING activation may exacerbate the local pro-inflammatory milieu, consequently supporting tumor progression [44].

Cancer-Associated Fibroblasts (CAFs) serve as structural and signaling hubs. cGAS-STING signaling pathway modulates their phenotypic and functional activation [45]. Activated CAFs influence tumor progression through two primary mechanisms: firstly, by remodeling the immune microenvironment to enhance its immunosuppressive properties; and secondly, by directly secreting various cytokines and matrix components to support tumor growth and invasion. Furthermore, CAFs themselves exhibit dual effects: while remodeled extracellular matrix (ECM) provides structural support, excessive matrix deposition can form a dense physical barrier that limits drug penetration and protects tumor cells [46].

Natural Killer (NK) cells are cytotoxic innate lymphocytes capable of directly lysing target cells [47]. Activation of the cGAS-STING pathway promotes the recruitment of NK cells to the tumor microenvironment and enhances their functional activation [48]. Activated NK cells exert significant anti-tumor effects by secreting IFN-γ and releasing cytotoxic granules to directly eliminate tumor cells. Although the cGAS-STING pathway typically serves as an upstream signal for NK cell activation, NK cells can form a positive feedback loop by secreting cytokines, modulating cellular metabolism, and altering tissue architecture. Studies have revealed that activated NK cells can promote the maturation of DCs, which serve as major mediators of STING signaling in the tumor microenvironment [49]. These mature DCs further activate NK cells through STING-mediated production of type IFN-I, establishing a positive feedback circuit [50].

T cells are among the most critical adaptive immune effector cells within the cGAS-STING pathway. Activation of the STING pathway can sustain the stemness of T cells and promote their differentiation into potent effector T cells that directly kill tumor cells [51]. STING activation can induce T cells to produce type IFN-I, a key molecule in antiviral immunity.

B cells display a dual role. STING activation can induce apoptosis in malignant B cells and promote anti-tumor humoral responses, yet in certain contexts, it may also support immunosuppressive regulatory B cells (Bregs) [52].

In summary, the functional outcome of cGAS-STING pathway activation is compartment-specific [53]. Activation within the immune cell compartment, particularly in DCs and NK cells, generally drives anti-tumor immunity, whereas activation within tumor cells or immunosuppressive stromal cells may inadvertently promote tumor progression. This spatial compartmentalization underscores the necessity of cell type‑specific targeting strategies for therapeutic intervention.

#### Spatial regulation by physicochemical gradients

The spatiotemporal activity of the cGAS-STING pathway is further shaped by physicochemical gradients within the TME, most notably the heterogeneous distribution of oxygen and nutrients [17]. Hypoxia, a core driver of immunosuppression prevalent in solid tumors, especially in avascular core regions—profoundly impairs cGAS-STING signaling through multiple convergent mechanisms: (1) HIF-1α-Mediated Downregulation of cGAS Expression: Hypoxia stabilizes Hypoxia-Inducible Factor-1α (HIF-1α), which transcriptionally represses key components of the cGAS-STING axis. For instance, HIF-1α induces specific microRNAs (miRNAs) that target and degrade mRNA of NCOA3, a transcriptional coactivator essential for cGAS expression. This reduction in cGAS protein levels diminishes the cell’s capacity to sense cytosolic dsDNA [54]. (2) Reduction of cGAS-Stimulating DNA Ligands: The hypoxic microenvironment also limits the availability of cytosolic dsDNA. Hypoxia can promote RNASEH2A-mediated degradation of cytosolic DNA and suppress the formation of cGAS-activating micronuclei via the SETDB1-TRIM28 complex, thereby depleting the ligand pool for cGAS activation [55]. (3) Suppression of Immune Effector Function: Hypoxia directly inhibits the function of DCs and T cells [56]. Consequently, even if the STING pathway is activated under these conditions, its downstream immunostimulatory output is severely attenuated. Simultaneously, hypoxia fosters the recruitment and immunosuppressive activity of regulatory cells, further compromising effective STING-mediated immunity.

Nutrient gradients and metabolic competition are another crucial factor through which the TME influences STING signaling [57, 58]. As solid tumors expand rapidly, their volume soon outstrips the perfusion capacity of the nascent vasculature, leading to severe hypoxia and nutrient deprivation within the tumor, particularly in its core region. This process not only generates a pronounced nutrient gradient from the periphery to the core but also drives intense metabolic competition among TME components—such as tumor cells and infiltrating immune cells—for limited resources [59]. The concentrations of key nutrients, including glucose, glutamine, amino acids, and lipids, also exhibit a decreasing gradient from the periphery to the core. Glucose is the primary source of ATP for cells. In the TME core region, severe glucose deprivation is one of the most direct and severe challenges faced by tumor cells. Researchers have found that intervening in lipid metabolism-related signaling to reshape the TME can impede colorectal cancer progression, thereby demonstrating that dysregulated lipid metabolism in colorectal cancer cells serves as a key modulator of the TME [60]. Accompanying nutrient depletion is the accumulation of metabolic waste products. For example, enhanced glycolysis leads to excessive lactate production and accumulation, lowering the pH in the tumor core region below physiological levels. The acidic environment not only suppresses antitumor immune cell function but also promotes tumor cell invasion and matrix remodeling [61]. Viewing the tumor as a dynamic metabolic ecosystem and regarding metabolic intermediates as dynamic signaling molecules facilitates a deeper understanding of how the TME shapes the signaling network of tumor cells through nutrient gradients and metabolic competition.

The spatial heterogeneity of the TME, driven by both cellular architecture and physicochemical gradients, leads to a functional compartmentalization of cGAS-STING pathway activity. At the invasive front, enriched with immune cells and adequate oxygenation and nutrients, STING activation can effectively drive anti-tumor immunity [62]. Conversely, in the immunosuppressive, hypoxic, and nutrient-deprived tumor core, STING signaling is often suppressed. This regional functional differentiation is key to explaining the variable efficacy of STING agonists across different tumor types and even within the same tumor.

### Temporal heterogeneity

The TME exhibits not only spatial heterogeneity but also undergoes dynamic evolution over time. Consequently, cGAS-STING pathway activity is highly plastic, demonstrating distinct temporal patterns across stages of tumor progression and therapeutic challenge [63].

#### Early stage

During early tumorigenesis, due to oncogene activation, DNA damage repair defects, etc., tumor cells begin to exhibit genomic instability. This leads to micronucleus formation and chromosomal abnormalities, resulting in the appearance of cytosolic dsDNA, which ultimately activates the tumor cell’s own cGAS-STING pathway intermittently or acutely [64]. This acute, high-intensity activation primarily drives the production of IFN-I, effectively recruiting and activating NK cells, DCs, and T cells to perform immune clearance of nascent tumor cells [65]. This constitutes a vital part of the immune surveillance mechanism.

#### Progressive stage

As the tumor progresses, the TME undergoes dramatic evolution, becoming highly heterogeneous [64]. Chronic, persistent activation of the cGAS-STING pathway may shift towards promoting tumor progression and metastasis. Chronic signaling can induce immunosuppressive TME. For instance, tumor cell-derived microparticles (T-MPs) can reprogram macrophages into immunosuppressive M2 phenotypes via the cGAS-STING pathway [66]. Concurrently, persistent interferon signaling may lead to T cell exhaustion and upregulation of immune checkpoint molecules like PD-L1. A key mechanism promoting metastasis involves chronic, low-intensity cGAS-STING signaling driven by chromosomal instability preferentially activating the non-canonical NF-κB pathway over the interferon pathway, thereby enhancing the invasive and metastatic capacity of tumor cells [37]. At this stage, the pathway transforms from an “immune sentinel” into an “accomplice” for tumor dissemination.

#### Therapeutic intervention stage

Under interventions like radiotherapy, chemotherapy, or immunotherapy, the TME is drastically remodeled, and the role of the cGAS-STING pathway becomes complex and time-dependent [67, 68]. In the initial phase of treatment, radiotherapy and chemotherapy can cause DNA damage and massive release of cytosolic DNA from tumor cells, triggering robust, acute cGAS-STING activation [69, 70]. This can promote antigen presentation and CD8⁺T cell infiltration, synergizing with immune checkpoint inhibitors (e.g., anti-PD-1 therapy) to enhance efficacy. Research indicates that post-radiotherapy TME remodeling has spatiotemporal characteristics, with early infiltration of B cells and CD4⁺T cells laying the groundwork for subsequent deep infiltration of CD8⁺T cells—a process associated with STING activation [71]. In the mid-to-late stages of treatment, sustained cGAS-STING signaling may once again foster an immunosuppressive environment. For example, radiotherapy alone can sometimes enhance the immunosuppressive function of MDSCs via this pathway [72]. Furthermore, tumor cells may adapt and develop resistance through mechanisms like downregulating STING expression (e.g., silencing by TGF-β). Clinical trials also suggest that systemic administration of STING agonist monotherapy may lead to the emergence of resistance.

Thus, along the temporal dimension, the function of the cGAS-STING pathway undergoes a transition from anti-tumor to pro-tumorigenic—evolving from an initial protective signal inducing immune clearance into a pathological pathway co-opted by the tumor to facilitate immune escape and malignant progression [73]. Harnessing the temporal plasticity of the cGAS-STING pathway will be key to unlocking its full potential as a therapeutic target in cancer.

## cGAS-STING-driven precision immunotherapy and artificial intelligence optimization

Artificial intelligence (AI) is a discipline of computer science that solves problems by emulating human thought processes [74]. The implementation of Artificial Intelligence contributes to disease prediction and monitoring, risk assessment of incidence or mortality, disease diagnosis and treatment, as well as health policy and planning [75]. AI can assist in modeling and predicting medical information. A growing body of research integrates radiology, pathology, genomics, and proteomics data to predict the expression levels of PD-L1, tumor mutational burden (TMB), and TME in cancer patients, or to forecast the likelihood of immunotherapy benefits and side effects [76]. The significant potential of the cGAS–STING pathway in lung cancer immunotherapy requires the optimization of its efficacy through multidimensional strategies [77]. We propose a precision Diagnosis–Intervention–AI Optimization (DIA) framework to achieve individualized treatment based on the “immunometabolic threshold.” This framework, via multidimensional diagnosis, stratified intervention, and dynamic AI-driven optimization, aims to maximize the therapeutic benefits of the cGAS–STING pathway in lung cancer treatment.

### Diagnostic stratification

Artificial intelligence enables precise patient stratification by integrating multi-omics data. AI algorithms can decode complex patterns from radiomics and pathological images, and fuse them with molecular data to identify clinically significant disease subtypes, discover non-linear relationships, and provide evidence for individualized treatment. For instance, the Sybil model analyzes low-dose CT images to predict individual lung cancer risk without requiring manual annotations or additional clinical data inputs [78]. Stratified diagnosis integrates multidimensional data, including genomic, transcriptomic, and proteomic profiles, to quantify STING activity and TME characteristics, achieving the identification of the patient’s specific “functional window,” with detailed detection dimensions presented in (Table 1).

**Table 1:** Precision diagnostic stratification framework guided by the cGAS-STING pathway

Detection Dimension	Key Technology	Predictive Marker	Clinical Significance
Liquid Biopsy	ddPCR/LC-MS/MS NGS fragmentomics + AI analysis	mtDNA, cGAMP levels AI-integrated cfDNA multi-feature profile	Dynamic monitoring of “systemic immune status”
Radiomics	¹⁸F-FDG PET-CT Dedicated probe PET/MRI AI-based radiomics	SUVmax and other metabolic parameters ⁶⁸Ga-DOTA-diABZI uptake rate AI-extracted texture/heterogeneity features	Spatial localization of “cold/hot tumors” and pathway activity
Tissue multi-omics	Multiplex immunofluorescence/digital pathology AI Spatial transcriptomics/proteomics Multi-omics data-integrated AI model	CD8⁺/FoxP3⁺ and other spatial ratios IRF3/NF-κB1 pathway activity profile Composite functional subtype signature	Dynamic monitoring of “systemic immune status”

### Targeted intervention

**Fig. 2:**
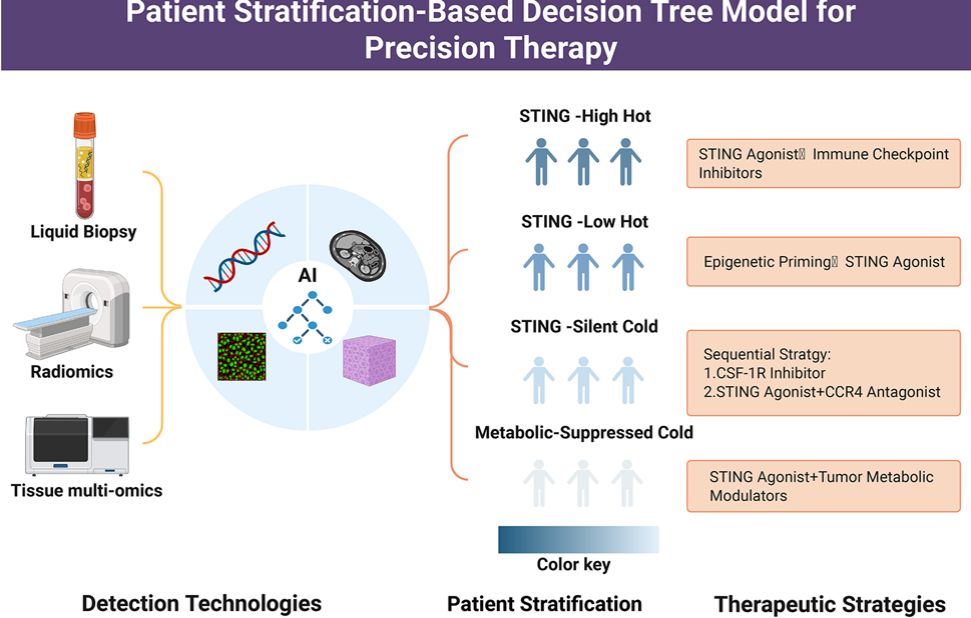
The DIA framework for cGAS–STING-targeted precision therapy. The workflow outlines the diagnosis–Intervention–AI Optimization (DIA) framework, which comprises three core components: Multimodal Diagnostics (liquid biopsy, radiomics, and tissue multi-omics), AI-powered patient stratification into four subtypes, and targeted intervention strategies. Following treatment initiation, dynamic monitoring data are fed into an AI optimization engine to adjust regimens in real time, forming a closed-loop precision therapy system. The color key on the right indicates the level of immune activity, ranging from low (dark blue) to high (light blue)

The decision tree, a classical machine learning algorithm, performs classification by constructing a tree-like decision structure and has been successfully applied in diverse scenarios [79]. Our proposed decision tree model classifies patients into four subtypes—STING-High Hot, STING-Low Hot, STING-Silent Cold, and Metabolic-Suppressed Cold—based on liquid biopsy, radiomics, and tissue analysis. Guided by this stratification, it formulates combination strategies with the core principle of modulating STING activity into the therapeutic window within the “immunometabolic threshold.” **(**Fig. 2**)**.

To enable precise implementation of the aforementioned stratified intervention strategies, innovations in delivery technologies serve as critical enablers. Smart responsive nanocarriers can achieve spatiotemporally controlled drug release based on biochemical or physical characteristics of the TME, thereby enhancing efficacy and reducing systemic toxicity. A variety of promising delivery systems have emerged: pH-responsive nanoparticles release cGAMP in the acidic TME (pH < 6.8) targeting the tumor core. There are also enzyme-responsive nanocarriers and hypoxia-responsive nanocarriers [80, 81].CAF-targeted liposomes co-deliver STING agonists and TGF-β inhibitors, simultaneously disrupting physical and immune barriers [82].Tumor-penetrating peptide-modified systems utilize iRGD peptide-modified nanocarriers to target αv integrins and neuropilin-1, enhancing tumor tissue penetration and cellular uptake for co-delivery of chemotherapeutic agents and immune agonists [83].These technologies, by intelligently responding to TME features or external guidance, significantly improve treatment precision and efficacy. Some have advanced to preclinical or early-stage clinical studies, providing essential tools for future intratumoral precision immunomodulation.

### AI-driven therapy optimization

AI has important value in predicting lung cancer treatment response and optimizing treatment plans [84]. By analyzing sequential medical imaging data, AI can track tumor dynamic changes and predict treatment effects and patient survival. Integrating multimodal data, AI models can predict STING activity, optimize combination regimens and treatment timing, and dynamically adjust to maximize efficacy and avoid toxicity [85]. Respond in real-time to TME evolution, dynamically adjusting treatment combinations and timing. Workflow **(**Table 2**)**: Patient multi-omics data input → AI model stratification → Initiate initial treatment → Dynamic monitoring (liquid biopsy/imaging) → AI engine adjusts plan → Feedback to clinical execution.

**Table 2:** AI-driven core modules for treatment optimization

AI Module	Input Data	Output Decision	Clinical Validation
Efficacy Prediction Model	Genomic (STING mutation/methylation) Transcriptomic (immune/metabolic signatures) Radiomics (PET heterogeneity)	STING Activity Score + Optimal Combination Regimen	AUC = 0.92 (TCGA-NSCLC cohort)
Dynamic Optimization Engine	Longitudinal liquid biopsy (mtDNA/cGAMP trend) Real-time imaging (tumor volume/metabolic change)	Adjustment of drug dose/timing	Model increased ORR by 35% (retrospective study)

### Challenges and future perspectives of AI-integrated precision therapy

AI shows broad application in lung cancer screening, diagnosis, staging, treatment planning, and prognosis prediction, demonstrating great potential in improving diagnostic accuracy and treatment efficacy, marking a significant shift in cancer treatment models [86]. The DIA framework proposed in this article offers a novel paradigm for integrating cGAS-STING pathway activity with artificial intelligence to advance precision immunotherapy in lung cancer. However, translating this framework from theory to broad clinical application still faces a series of challenges.

First, there are challenges related to the clinical translation of the cGAS-STING pathway itself. Systemic administration of STING agonists may trigger systemic inflammatory responses, limiting their therapeutic window. Moreover, precisely delivering agonists to the tumor site and achieving spatiotemporally accurate regulation according to the immunometabolic threshold theory, so as to avoid potential tumor-promoting effects, remains an unresolved technical bottleneck. The high spatiotemporal heterogeneity within tumors also means that single-site biopsies or detection may not accurately reflect the overall immune status of the tumor, posing difficulties for precise diagnostic stratification [87].

Second, challenges exist in the integration of AI technology. As mentioned earlier in this article, the performance of AI models heavily depends on high-quality, large-scale training data. Currently, there is a lack of standardized public datasets that integrate multi-omics, imaging, treatment response, and long-term follow-up data. Data heterogeneity, annotation errors, and quality control issues with real-world data present significant obstacles to model generalizability [88]. Additionally, the *black box* nature of AI models makes their decision-making processes difficult to interpret, which may hinder trust and adoption by clinicians [89]. On the implementation level, practical hurdles such as limited medical resources, insufficient training of specialized personnel, and technical barriers in integrating existing hospital information systems with AI algorithms also need to be overcome.

Despite these challenges, the prospects remain promising. Future directions should focus on establishing cross-institutional collaborations to build large, standardized databases encompassing diverse ethnic groups, lung cancer stages, and treatment modalities; developing interpretable AI models to enhance clinical credibility; and creating programming interfaces that seamlessly integrate with electronic health records to promote the incorporation of AI into clinical workflows. At the same time, continuous attention must be paid to patient privacy, data security, and ethical oversight [90]. Through multidisciplinary collaborative efforts to overcome these barriers, we can hope to truly realize individualized treatment based on the dynamic activity of the cGAS-STING pathway, ultimately improving survival outcomes for lung cancer patients.

## Conclusion and outlook

The dual functionality of the cGAS-STING pathway in lung cancer underscores the central regulatory role played by the spatiotemporal heterogeneity of the TME [91]. This situational dependence characteristic requires us to abandon a uniform treatment approach and instead focus on precise and dynamically adjustable intervention paradigms. This review systematically elaborates the context depends on temporal dimensions. Building on this foundation, we propose an integrated DIA framework. Its core involves leveraging multidimensional diagnostics to quantify STING activity and TME features, thereby enabling precise patient stratification. Guided by the immunometabolic threshold hypothesis, stratified therapeutic strategies are then designed, and artificial intelligence models are employed for dynamic outcome prediction and regimen optimization. Moreover, the marked divergence in STING pathway integrity between NSCLC and SCLC underscores the necessity of histology‑based stratification in future clinical trials of STING‑targeted agents, moving beyond a unified therapeutic paradigm toward subtype‑specific precision strategies [92].

Future research should aim to quantitatively define this threshold and develop real-time monitoring tools, ultimately facilitating dynamically adjusted therapy. In summary, a deeper understanding of TME-driven spatiotemporal regulation of the cGAS-STING pathway, combined with the implementation of innovative frameworks, holds significant potential to overcome current barriers of immunotherapy resistance in lung cancer and to translate this potent yet complex immune pathway into sustained clinical survival benefits.

## Data Availability

No datasets were generated or analysed during the current study.
